# Metabolomics-based exploration of the pathogenesis of breast cancer-related fatigue and progress of traditional Chinese medicine intervention

**DOI:** 10.3389/fonc.2026.1702291

**Published:** 2026-06-29

**Authors:** Yidi Jiang, Chaofan Zhao, Qi Qiu, Qin Feng, Le Yang, Zhengcan Zhou, Mingqian Jia, Huiru Liao, Qinnan Li, Ye Sun, Jianjun Gu, Bo Yang, Ning Zhang, Hui Sun

**Affiliations:** 1 State Key Laboratory of Integration and Innovation of Classic Formula and Modern Chinese Medicine, National Chinmedomics Research Center, National TCM Key Laboratory of Serum Pharmacochemistry, Metabolomics Laboratory, Department of Pharmaceutical Analysis, Heilongjiang University of Chinese Medicine, Harbin, China; 2 Shandong Key Laboratory of Gelatin-Based Drug Research and Development, National Engineering Technology Research Center for Gelatin-based TCM, Shandong Provincial Engineering Research Center of Gelatin-Based Traditional Chinese Medicine for Technology Innovation and Application, Dong’e Ejiao Co., Ltd., Liaocheng, China; 3 State Key Laboratory of Dampness Syndrome, The Second Affiliated Hospital, Guangzhou University of Chinese Medicine, Guangzhou, China

**Keywords:** breast cancer, cancer-related fatigue, herb, metabolomics, traditional Chinese medicine

## Abstract

Breast cancer-related fatigue is a persistent and subjective sense of tiredness caused by either the cancer itself or its treatments. This fatigue is disproportionate to recent physical activity and significantly interferes with daily life. The exact pathogenesis of Breast cancer-related fatigue remains unclear. Breast cancer cells induce metabolic abnormalities through reprogramming. This disruption in metabolic balance—particularly in various systems—may contribute to Breast cancer-related fatigue by compromising the body’s overall metabolic homeostasis, all while supporting the biosynthetic demands of cancer cell proliferation. Current pharmacological treatments for cancer-related fatigue have demonstrated limited and inconsistent efficacy. These treatments are often hindered by a narrow drug target profile, high rates of adverse effects, and the complex mechanisms underlying fatigue, particularly those related to metabolic changes. Traditional Chinese medicine, with its multi-target modulation and lower toxicity, holds significant potential as long-term adjunctive or alternative treatments for Breast cancer-related fatigue. This article reviews the current understanding of the metabolic causes of Breast cancer-related fatigue and explores the role of traditional Chinese medicine interventions, offering new insights into potential targeted therapies for this condition.

## 1 Introduction

The incidence of breast cancer has risen dramatically worldwide over the past two decades. Although advances in chemotherapy, radiotherapy, and targeted therapies have led to a decline in breast cancer mortality (1), patients often experience symptoms such as reduced physical functioning, impaired mental health, and fatigue both before and after diagnosis. These symptoms are typically exacerbated during and following treatment (2, 3). Cancer-related fatigue (CRF) is a multifaceted symptom characterized by a persistent feeling of physical, emotional, and mental exhaustion that lasts for more than six months and does not improve with rest or sleep (4, 5), causing a more serious negative impact on the patient’s quality of life.

The pathogenesis of CRF remains largely unexplored. A substantial body of research suggests that metabolic reprogramming is a key feature of breast cancer progression. Cancer cells alter their metabolic profiles to meet the increased demands of cell proliferation. For example, lipid metabolism is significantly upregulated throughout the body in breast cancer patients, and there is a disruption in amino acid metabolism, including that of leucine and glutamine (6, 7). Additionally, chemotherapy drugs like paclitaxel indiscriminately attack rapidly dividing cells, affecting the uptake of glucose, amino acids, and other nutrients in normal, non-cancerous cells. This can lead to metabolic dysfunction, a major factor contributing to the development of CRF and other complications in breast cancer (8) (9),. Existing studies suggest that the mechanism underlying fatigue may involve a combination of metabolic disorders and other biological processes, indicating that CRF arises from the interplay of dysfunctional metabolic pathways across multiple systems and the overall disturbance of the body’s physiological state during cancer progression or treatment.

Metabolomics, an emerging field of research, aims to comprehensively analyze and quantify the small-molecule metabolites (molecular weight under 1500 Da) in an organism under specific physiological or pathological conditions, and to explore their relationship with biological processes. These metabolites are the final products of ongoing metabolic pathways within and outside of cells, found in human tissues or biological fluids (10). Metabolomics also serves as a bridge between genotype and phenotype, reflecting dynamic changes in endogenous metabolites during pathological states or following drug interventions (11–13). In previous studies, Feng et al. used an untargeted metabolomics approach to identify significant differences in the levels of compounds like cystine, γ-glutamylglutamine, and maleate in the serum of CRF patients compared to healthy volunteers (14). This suggests that metabolomics holds promise as a valuable tool for identifying diagnostic biomarkers, elucidating molecular mechanisms, and discovering potential therapeutic targets for CRF in breast cancer (7, 15, 16).

While existing therapies such as methylphenidate and physical exercise have been shown to alleviate CRF symptoms (17), they are limited by single-target mechanisms, significant side effects, or short-term efficacy (18, 19).In contrast, traditional Chinese medicines (TCM)—including individual herbs, TCM compounds, extracts, and their active ingredients—offer unique advantages due to their multi-target effects, low toxicity, and fewer side effects. These properties make TCM a promising option for the adjuvant treatment of CRF symptoms associated with systemic metabolic abnormalities (20). Several studies have demonstrated that certain TCM and their key active ingredients can reduce fatigue in breast cancer patients by regulating metabolic processes and mitigating treatment side effects (21, 22). For example, astragali polysaccharides show potential in addressing this clinical gap (23).

This review aims to explore the pathogenesis of breast cancer-related CRF and the progress of TCM interventions, based on current metabolomics findings. This exploration holds significant potential for advancing the use of TCM in the treatment of CRF in breast cancer patients.

## 2 High incidence and unknown mechanism of CRF in breast cancer

CRF is a persistent and unrelieved sensation of fatigue experienced by cancer patients during or after treatment, which cannot be adequately alleviated even with sufficient rest. Recent studies indicate that breast cancer is the most prevalent cancer among women globally. As awareness of breast cancer screening increases and treatment methods improve, the survival rates of breast cancer patients have improved. However, this progress has been accompanied by a rise in the prevalence of CRF (24), which disrupts patients’ normal physical activities and significantly diminishes their quality of life. Reports suggest that between 58% and 94% of breast cancer patients experience fatigue during treatment, with 56% to 95% reporting fatigue-related symptoms following adjuvant chemotherapy (25).

Currently, our understanding of the etiology of CRF remains limited. Fatigue can be classified as either central or peripheral. Central fatigue is characterized by diminished motivation and execution of movement due to processes in the central nervous system (CNS), while peripheral fatigue refers to impaired signaling at the neuromuscular junction (NMJ) or reduced force production by skeletal muscles during exercise performance (26). Numerous studies have shown that macroscopic metabolism is altered in breast cancer patients compared to healthy individuals, and this dysregulated metabolism interacts with cellular signaling pathways, affecting CNS, NMJ, and peripheral muscle function, thereby contributing to both breast cancer progression and CRF development (27).

The susceptibility of breast cancer patients to CRF is influenced by multiple factors. Carlos et al. employed the Perceived Effort Score, Finger Tapping Test, and Brief Fatigue Scale to evaluate CNS impact in CRF patients. Their results indicated increased perceived effort scores among all patients capable of completing the finger tapping test, confirming that CRF symptoms originate centrally (26). Arya et al. found that fatigue in breast cancer patients correlates with various functional changes in brain regions and networks associated with executive functions, including memory, planning, and attention. Patients were found to be at heightened risk for fatigue even before initiating treatment (28). Additionally, the integrity of the blood-brain barrier may be compromised by chemotherapeutic agents used in breast cancer, contributing to the central pathology associated with CRF (29, 30).

On the peripheral level, Tamara et al. demonstrated that elevated fatigue levels in breast cancer survivors significantly correlate with increased mitochondrial oxygen consumption and heightened glycolytic activity in skeletal muscle, indicating that abnormal cellular energy metabolism plays a crucial role in the development of CRF (31). Furthermore, decreased muscle strength, poor bone health, disrupted basal metabolic activity, and weight gain associated with breast cancer and treatment can independently or synergistically induce or exacerbate CRF in patients (32, 33).

Currently, a systematic analysis of the pathological mechanisms underlying CRF is urgently needed, as well as targeted treatment plans that warrant further research. This urgency arises from the high prevalence of CRF among breast cancer survivors, the chronic nature of the condition, and the limitations of existing therapeutic approaches. Therefore, overcoming current research bottlenecks, precisely analyzing the pathogenesis of breast cancer-related CRF, identifying key targets, and developing effective intervention strategies targeting the CNS-NMJ-muscle network are essential steps to improve the quality of life for breast cancer patients suffering from CRF.

## 3 Abnormal metabolism in breast cancer contributes to fatigue

Breast cancer patients experience a cluster of CRF symptoms from before to after treatment, characterized by persistent and subjective fatigue that severely impacts their quality of life (34). As a complex disease primarily marked by metabolic alterations, breast cancer drives tumor development by remodeling systemic metabolic profiles to meet the metabolic demands of cancer cell proliferation (35). This disruption profoundly affects both central and peripheral metabolism, contributing to the development of CRF. Metabolomics, a powerful tool for identifying altered biomarkers and metabolic pathways in cancer, is increasingly used to investigate the pathogenesis of CRF in breast cancer (7, 36). The rapid advancement of modern high-throughput metabolomics enables researchers to systematically analyze patient metabolites and small molecules in tissues, providing valuable insights into the underlying mechanisms of CRF (37). The available metabolomics data on breast cancer are summarized in **Table 1**.

**Table 1:** Existing metabolomics studies in breast cancer.

Breast cancer subtypes	Sample	Analytical platform	Differential metabolites and trend	Associated with fatigue (Propose)	Related metabolic pathway	Year	References
Breast cancer	Serum	HPLC-MS/MS	**↓: Tryptophan**	Neurotransmitter imbalance - Central fatigue	Tryptophan metabolism	2023	(42)
Breast cancer	Serum	UPLC-Triple TOF-MS	↓: Furcelleran, N-methylniacinamide, **4-hydroxy-1H-indole-3-acetonitrile**, β-glyceryl phosphate, geranyl acetoacetate, styrene oxide, **serotonin**, and synephrine acetone	Neurotransmitter imbalance - Central fatigue	Histidine metabolism, phenylalanine, tyrosine, and tryptophan biosynthesis, taurine and hypotaurine metabolism, phenylalanine metabolism, and β-alanine metabolism	2022	(44)
Breast cancer	Serum	MS	↑: Hexose **↓: Glutamic acid**, lysophatidylcholine, diglycerides, and aspartic acid	Disruption of mitochondrial metabolism-Insufficient energy supply to the muscles	Glutamate metabolism, aspartic acid metabolism, lipid metabolism, glucose metabolism	2024	(55)
Breast cancer	Serum	LC-MS	**↑: Oxpcs** and **SMs**	Reduced efficiency of neurotransmitter release-Central fatigue	Lipid metabolism	2023	(56)
Breast cancer	Serum	LC-MS	↑: Lysophosphatidylethanolamine, glutarylcarnitine, and **sphingomyelin** **↓: Ceramides** and **LPC**	Damage to synaptic structures- Impairment of NMJ signal transmission	Sphingolipid metabolism, phospholipid metabolism, and fatty acid β-oxidation	2015	(79)
Breast cancer	Serum	LC-MS/MS	**↑:3-methylhistidine**, aminobutyric acid, hydroxyproline, and acylcarnitine **↓: Tryptophan, serine**, and **sphingolipid**	Myosin breakdown–Loss of muscle mass/Neurotransmitter imbalance-Central fatigue	Fatty acid metabolism, SM, PC, and lysophospholipid metabolism, serine, tryptophan, hydroxyproline, and histidine metabolism	2014	(91)
TNBC	Serum	NMR	↑:2-aminobutyric acid, N, N-dimethylglycine, asparagine, proline, and ornithine ↓: Alanine and **glucose**	Disruption of mitochondrial metabolism-Insufficient energy supply to the muscles	Glycine, serine, and threonine metabolism, valine, leucine, and isoleucine biosynthesis, and alanine, aspartic acid, and glutamic acid metabolism	2021	(96)
Breast cancer	Serum	NMR	**↑: Lactic acid**, glutamic acid, lysine, and alanine **↓: Glucose** and **pyruvate**	Disruption of mitochondrial metabolism-Insufficient energy supply to the muscles	Glycolysis/gluconeogenesis, glutamate metabolism, alanine/aspartic acid/glutamate metabolism, lysine degradation, lipid metabolism	2017	(98)
TNBC	Serum	LC-MS	**↑: L-glutamate** **↓: Citrate**, creatine, creatinine	Disruption of mitochondrial metabolism-Insufficient energy supply to the muscles	Tricarboxylic acid cycle, alanine, aspartic acid, and glutamate metabolism	2024	(99)
Breast cancer	Plasma	MS	**↑: Serotonin, phenylacetylglycine**, cinnamic acid, and oxatin acid ↓:4-hydroxybenzoic acid and **indole sulfate**	Neurotransmitter imbalance-Central fatigue/Disruption of mitochondrial metabolism-Insufficient energy supply to the muscles	Ubiquinone pathway, biosynthesis of other terpene quinones, and tryptophan metabolism	2024	(43)
Non-metastatic breast cancer	Plasma	UHPLC-MS	**↑: Kynurenine** and **kynurenine/tryptophan ratios** ↓: **Serotonin**	Kynurenine pathway activation -Central fatigue	Tryptophan metabolism	2018	(54)
Breast cancer	Plasma	NMR	**↑**: Glutamic acid, **lactic acid**, NAG, hydroxybutyric acid, lipid ↓: glycine	Disruption of mitochondrial metabolism-Insufficient energy supply to the muscles	Glutamine-glutamate metabolism, pyruvate metabolism, arginine-proline metabolism, glycine-serine-threonine metabolism, D-glutamate metabolism	2018	(97)
Breast cancer	Plasma	LC-QTOF-MS	↑: Hypoxanthine, **phosphatidylinositol**, 2-hydroxymyristic acid, and biliverdin ↓: Caprylic acid, sorbitol, and **allantoin**	Disruption of mitochondrial metabolism-Insufficient energy supply to the muscles	Fatty acid metabolism, inositol phosphate metabolism	2021	(108)
Breast cancer	Plasma	GC-EI-QTOF-MS	↑: Hypoxanthines, maltose, and 3-**phosphoglyceric acid** ↓: Dicarboxylic acid	Disruption of mitochondrial metabolism-Insufficient energy supply to the muscles	Purine metabolism and galactose metabolism	2024	(109)
Breast cancer	Breast tumor tissue	LC/MS	↑: Malic acid, butyrylcarnitine, histidine, isocitrate, hypoxanthine, and adenosine **↓: Glucose**	Insufficient energy substrates-Reduced energy supply to muscles	TCA cycle, purine and pyrimidine metabolism, fatty acid β oxidation, amino acid metabolism, glycolysis	2021	(8)
Breast cancer	Breast tumor tissue	NMR	**↑: Pyruvate, citric, succinic, fumarate**, glutamic acid, glyceryl phosphocholine, sphingomyelin, uric acid, inosine, and adenosine are elevated **↓: Leucine, isoleucine, valine**, phosphocholine, thymine	Competitive utilisation of energy substrates by tumours - Reduced energy supply to muscles/A reduction in amino acids that promote muscle protein synthesis - Impaired muscle growth	Glycolysis and TCA cycle, glutamate metabolism, choline metabolism (Kennedy pathway), lipid metabolism, antioxidant, and nucleotide metabolism	2020	(76)
ER+	Breast tumor tissue	UHPLC-Q Exactive Plus MS	**↑: Glycerophospholipids, sphingolipids**, free fatty acids, glycine, **serine**, and threonine	Damage to synaptic structures-Impairment of NMJ signal transmission	Lipid/fatty acid metabolism, nucleotide metabolism, glutamine metabolism, glycolysis and oxidative phosphorylation, glutathione metabolism	2022	(80)
Breast cancer	Breast tumor tissue	NMR/GC-qMS	**↑: Lactic acid, glutamic acid**, and taurine ↓: Valine	Insufficient energy substrates-Reduced energy supply to muscles	Lactate metabolism, glutamate metabolism, valine metabolism, aspartic acid metabolism, choline metabolism	2021	(100)
Breast cancer	Breast tumor tissue	UHPLC-MS/MS	**↑: Glutamic acid**, 2-hydroxyglutaric acid, β-alanine, hypoxanthine, and membrane phospholipids **↓: Glutamine** and free fatty acids	Insufficient energy substrates-Reduced energy supply to muscles	Glutamine metabolism, lipid metabolism, nucleotide metabolism, β-alanine metabolism	2016	(101)
Breast cancer	Breast tumor tissue	GC-TOF-MS	↑: Glycerin, **glutamine**, glucose-1-phosphate, and urea ↓: Arginine and carnitine are increased, and maltose and aspartic acid	Competitive utilisation of energy substrates by tumours-Reduced energy supply to muscles	Glutamine metabolism, urea cycle, phosphatidylethanolamine biosynthesis, glutathione metabolism, fatty acid metabolism, ammonia cycle, glycolysis, and gluconeogenesis	2020	(102)
Invasive ductal carcinoma, invasive lobular carcinoma, ductal carcinoma in situ	Urine	GC-MS	↑: Urea **↓: High vanillate**, 4-hydroxyphenylacetate, 5-hydroxyindole acetate	A reduction in DA breakdown products suggests a decrease in central DA levels – Central fatigue	Tyrosine metabolism, tryptophan metabolism	2009	(62)
Breast cancer	Urine	NMR/GC-qMS	↑: A-hydroxyisobutyric acid, **glutamine**, and hypoxanthine ↓: Betaine	Competitive utilisation of energy substrates by tumours-Reduced energy supply to muscles	Glutamine metabolism, hypoxanthine metabolism, and ketone body metabolism	2021	(100)
TNBC	Gastrocnemius	1H NMR	↓: Lysine, isobutyric acid, AMP, 3-hydroxyphenylacetic acid, **pyruvate**, and creatine	Insufficient energy substrates-Reduced energy supply to muscles	Pyruvate metabolism, glycolysis/gluconeogenesis, and glyoxylic acid and dicarboxylic acid metabolism	2022	(106)

The arrow symbols (↑/↓) indicate the upward or downward trends in the expression of differentially expressed metabolites across studies; differentially expressed metabolites marked in bold are potential biomarkers associated with breast cancer-related fatigue.

### 3.1 Central neurotransmitter imbalance in breast cancer contributes to fatigue

#### 3.1.1 Abnormalities in multiple metabolisms in breast cancer affect 5-HT synthesis and release

Studies have shown that the severity of fatigue in breast cancer patients is positively correlated with levels of depression, and that fatigue and depression are highly comorbid (38, 39). 5-HTergic neurons from the dorsal raphe nucleus project to the ventral tegmental area, where they influence the reward circuitry via 5-HT1A receptors, playing a significant role in regulating depressive behavioural states (40). 5-HT is primarily synthesised from free tryptophan in the blood, which crosses the blood-brain barrier and is catalysed by tryptophan hydroxylase and 5-hydroxytryptophan decarboxylase (41). Several metabolomic studies have found that both tryptophan levels and levels of the 5-HT metabolite 5-hydroxyindoleacetic acid are significantly reduced in the plasma of breast cancer patients (42–44). Several metabolomic studies have found that both tryptophan levels and levels of the 5-HT metabolite 5-hydroxyindoleacetic acid are significantly reduced in the plasma of breast cancer patients.

**Figure 1:**
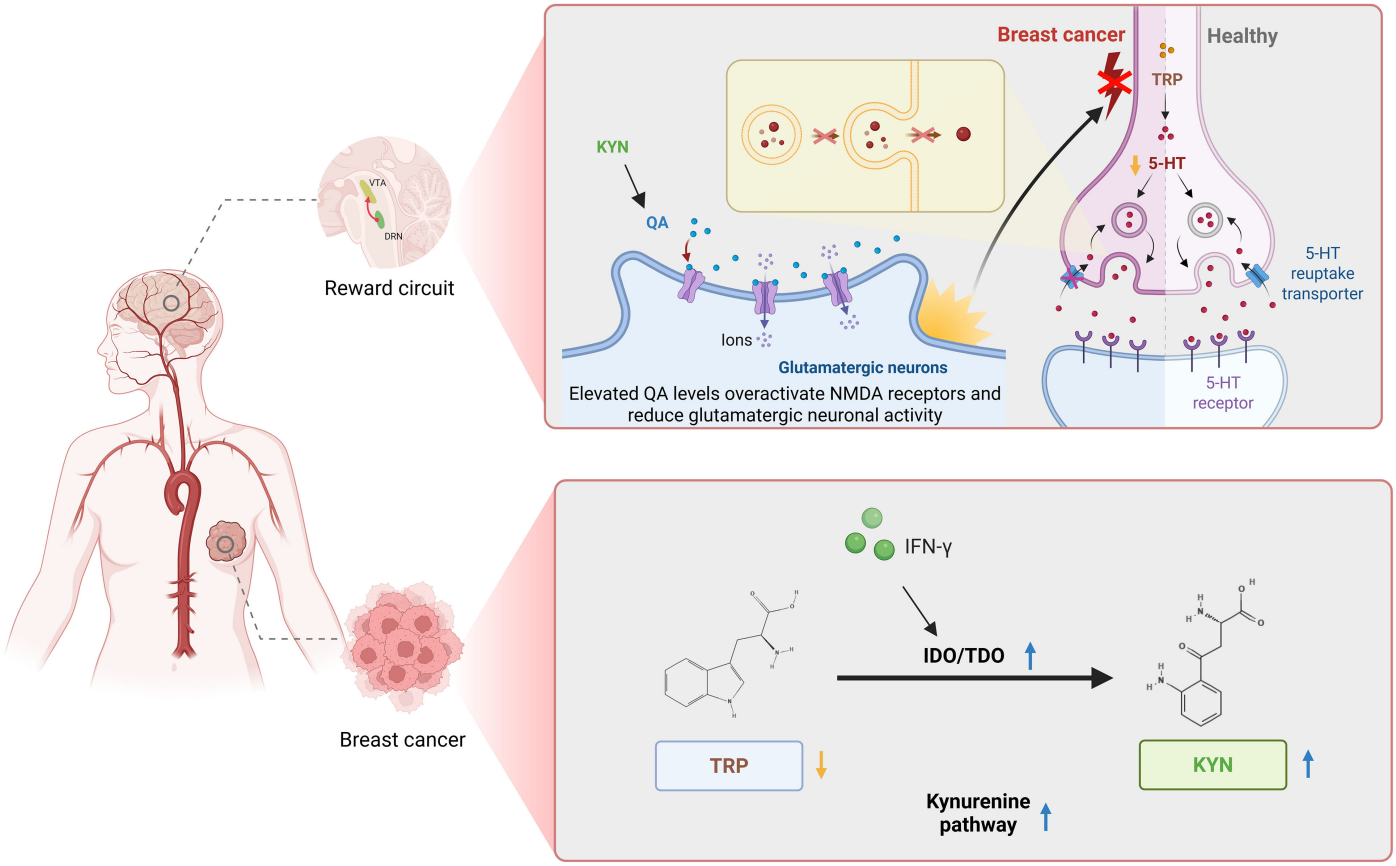
Schematic diagram of the mechanism by which decreased 5-hydroxytryptamine (5-HT) neuronal activity contributes to cancer-related fatigue (CRF) in breast cancer. Increased indoleamine 2,3-dioxygenase (IDO)/tryptophan 2,3-dioxygenase(TDO) expression in breast tumor tissues depletes tryptophan (TRP) and decreases 5-HT synthesis substrates in the central nervous system (CNS). TRP metabolism is skewed toward the kynurenine (KYN) pathway, resulting in an increase in KYN from the periphery to the center, resulting in quinolinic acid (QA) that over-activates N-methyl-D-aspartate (NMDA) receptors, decreases glutamatergic neuronal activity, and further affects 5-HT neuron firing and 5-HT release in targeted brain regions. Lipid metabolism remodeling associated with breast cancer may also reduce the efficiency of central neurotransmitter release. Created in https://BioRender.com.

Furthermore, elevated levels of indoleamine 2,3-dioxygenase shift tryptophan metabolism towards the kynurenine pathway. Indole-2,3-dioxygenase is the rate-limiting enzyme in the first step of tryptophan conversion to kynurenine; it catalyses the oxidative cleavage of the pyrrole ring of tryptophan to generate kynurenine (45). In an inflammatory context, IFN-γ binds to its receptor to activate the JAK/STAT1 signalling pathway, and upon translocation of the STAT1 dimer to the cell nucleus, it binds to the interferon-stimulated response element and the γ-activation site in the promoter region of the indoleamine 2,3-dioxygenase gene, thereby enhancing the transcriptional level of the indoleamine 2,3-dioxygenase gene (46). Consistent with the observation that the tumour microenvironment and inflammatory factors can increase indole-2,3-dioxygenase expression levels, numerous studies have demonstrated elevated indole-2,3-dioxygenase expression in tumour tissues of breast cancer patients, driving tryptophan metabolism towards the kynurenine pathway (47, 48). Changes in blood kynurenine levels also affect the central nervous system. Elevated levels of quinolinic acid, a metabolite of kynurenine, can hyperactivate NMDA receptors and reduce the activity of glutamatergic neurons (49). 5-HT neurons located in the dorsal raphe nucleus receive modulatory inputs from glutamatergic neurons, thereby influencing the firing and 5-HT release of 5-HT neurons in both local and distal target brain regions (50). This suggests that, in the context of breast cancer, the shift in tryptophan metabolism towards the kynurenine pathway affects the synthesis and transmission of central 5-HT, thereby contributing to the onset of breast cancer-related fatigue symptoms (**Figure 1**). 

At the same time, 5-HT release is closely linked to lipid metabolism. During the process of exosomal release, lipids provide the physical barrier, fluidity, and fundamental mechanical stability of the membrane, serving as the platform for SNARE protein complex-mediated membrane fusion events (51). Given the specific site of breast cancer development, a key characteristic of breast cancer cells is their enhanced ability to absorb fatty acids, leading to systemic lipid metabolism abnormalities in patients (52). Brain lipids are primarily composed of cholesterol, phospholipids, and sphingolipids. Their unique lipid composition and dynamic regulation are crucial for maintaining synaptic integrity and facilitating effective neurotransmitter release (53, 54). Serum metabolomics results indicate significantly reduced levels of lysophosphatidylcholine and glycerol diesters in breast cancer patients (55, 56), suggesting that lipid metabolic reorganisation in breast cancer may contribute to the development of cancer-related fatigue by reducing the efficiency of neurotransmitter release in the central nervous system.

#### 3.1.2 Reduced DA synthesis under breast cancer affects motor motivation generation and motor behavioral command transmission

Dopamine plays a key role in central motor neural circuits, influencing motor initiation, force modulation, and sustained activity; whilst participating in motor-related decision-making, it is also responsible for generating the motivation to move (57). A decrease in its levels or functional impairment can lead to difficulties in initiating movement and increased feelings of fatigue. The dopaminergic neurons of the ventral tegmental area are the primary source of central dopamine; the nigrostriatal pathway controls motor function, whilst the motivational circuit sends projections to the nucleus accumbens to generate motivational control (58–60). L-tyrosine, the precursor for dopamine synthesis, is transported from the bloodstream into the brain via the blood-brain barrier. Within the brain, L-tyrosine is converted into L-3,4-dihydroxyphenylalanine (DOPA) by the enzyme tyrosine hydroxylase, and subsequently into dopamine via the action of dopa decarboxylase. Following a series of enzymatic reactions in the body, dopamine is metabolised into biologically inactive high-vanillic acid. High-valeric acid is filtered out via the kidneys and ultimately excreted from the body in urine.

Several metabolomics studies have indicated that breast cancer patients may experience reduced central DA synthesis. Wojciech et al. conducted a study analyzing serum samples from triple-negative breast cancer (TNBC) patients and healthy controls using 1H NMR metabolomics. They found that serum tyrosine levels were significantly lower in TNBC patients compared to healthy individuals (61). This change may be associated with increased tyrosine consumption resulting from tumour metabolic reprogramming, reflecting abnormalities in the body’s amino acid metabolism under cancer conditions. Nam et al. found reduced levels of homovanillic acid in the urine of breast cancer patients (62), indirectly reflecting impaired dopamine synthesis in the brain. When dopamine production is reduced, patients experience an imbalance between motivation and behavioural execution; they are reluctant to engage in voluntary activities and exert less effort to obtain rewards, which is consistent with the clinical presentation of breast cancer patients (63). At the same time, patients are unable to translate their intentions into effective action, or they quickly feel overwhelmed during the course of action, thereby exacerbating their subjective perception of fatigue.

#### 3.1.3 Disruption of the blood-brain barrier by chemotherapy increases susceptibility to CRF in breast cancer patients

Chemotherapy is central to breast cancer treatment, being utilized across various stages and often in conjunction with targeted therapies and immunotherapies. This multimodal approach significantly enhances patient survival rates (64). However, the side effects of chemotherapy, particularly increased fatigue, should not be overlooked, especially toward the end of treatment (65). Paclitaxel (PTX), a key chemotherapeutic agent, inhibits mitosis in cancer cells by stabilizing microtubules and preventing their depolymerization. It is widely used in various cancers, including breast cancer, and possesses strong antiangiogenic properties (66, 67). Chetan et al. demonstrated that PTX treatment induces endothelial cell senescence, disrupting the blood-brain barrier (BBB). Under normal physiological conditions, the BBB serves as a critical barrier, providing nutritional support for neuronal connectivity and collaboration. It regulates substance transport and maintains homeostasis, ensuring efficient synaptic signaling and information integration, which are essential for cognitive functions. These mechanisms work together to preserve the accuracy and sustainability of central nervous system (CNS) functions. However, when the BBB is compromised, blood-derived factors can infiltrate the brain parenchyma, leading to neuroinflammation, synaptic dysfunction, and neurodegeneration (68). These disruptions impair cognitive function, increase susceptibility to fatigue in breast cancer patients, and negatively impact their ability to tolerate subsequent treatments, ultimately reducing their overall quality of life.

Chemotherapy drugs exert multiple effects contributing to central fatigue in breast cancer patients. Studies have shown that treatment with PTX can promote the release of pro-inflammatory cytokines, which in turn increase the permeability of the BBB. This creates a vicious cycle in the CNS characterized by “increased levels of pro-inflammatory cytokines → increased permeability of the blood-brain barrier → increased levels of pro-inflammatory cytokines,” thereby exacerbating neuroinflammation (69). Metabolomics studies have indicated that PTX treatment leads to elevated levels of serum secondary bile acids, particularly deoxycholic acid (DCA), and a significant decrease in bound bile acids, such as tauroursodeoxycholic acid (TUDCA) (70). Circulating bile acids can cross the BBB and enter the CNS via passive diffusion or bile acid transport proteins (71). Under normal circumstances, TUDCA binds to the Takeda G protein-coupled receptor 5 expressed in microglia, leading to increased intracellular cyclic AMP (cAMP) levels. This process induces the expression of anti-inflammatory markers, mitigating neurological damage (72). However, reduced TUDCA levels diminish this protective effect, adversely affecting the basal ganglia and thalamus in motor initiation, motivation, reward processing, and sensory information integration. Consequently, this results in motor retardation, anhedonia, and heightened fatigue. These findings suggest that the chemotherapeutic agent paclitaxel may exacerbate fatigue symptoms in patients through dual mechanisms: inflammation-mediated BBB disruption and metabolic reprogramming.

### 3.2 Neuromuscular junction dysfunction in breast cancer contributes to fatigue

The NMJ is a specialized synapse that serves as a “bridge” between motor neurons and skeletal muscle fibers. It converts electrical impulses from motor neurons into action potentials within muscle fibers (73). When the action potential reaches the axon terminal, it triggers an influx of calcium into the presynaptic membrane, leading to the release of acetylcholine (ACh) into the synaptic cleft. ACh binds to nicotinic acetylcholine receptors (nAChR) on the postsynaptic membrane, triggering sodium influx, which depolarizes the membrane and generates the endplate potential (EPP), ultimately inducing muscle fiber contraction (74). This process is finely regulated by presynaptic calcium channels, rapid degradation of ACh by acetylcholinesterase, and the presence of high-density receptors and sodium channels on the postsynaptic membrane. Disruption of this balance in pathological conditions results in impaired NMJ signaling.

A deficiency in phosphorylcholine can disrupt neuromuscular signaling through three key mechanisms. First, phosphocholine is crucial for the synthesis of cytidine diphosphate choline (CDP-choline), a key precursor in ACh biosynthesis. Inadequate CDP-choline production leads to reduced ACh synthesis in presynaptic neurons, diminishing the neurotransmitter reserve at motor nerve endings and impairing the conversion of electrical signals to chemical signals. Second, the reduced ACh reserve limits the availability of ACh for loading into synaptic vesicles and release. This results in a reduced amount of ACh in each vesicle and potentially fewer vesicles containing ACh, decreasing the probability of neurotransmitter release upon action potential arrival. Consequently, insufficient ligand binding to AChRs on the endplate membrane causes a characteristic attenuation of the EPP amplitude, making it difficult to reach the threshold for skeletal muscle contraction even with high-frequency nerve impulses (75). Additionally, the frequency and conduction velocity of myofibrillar action potentials decrease simultaneously.

**Figure 2:**
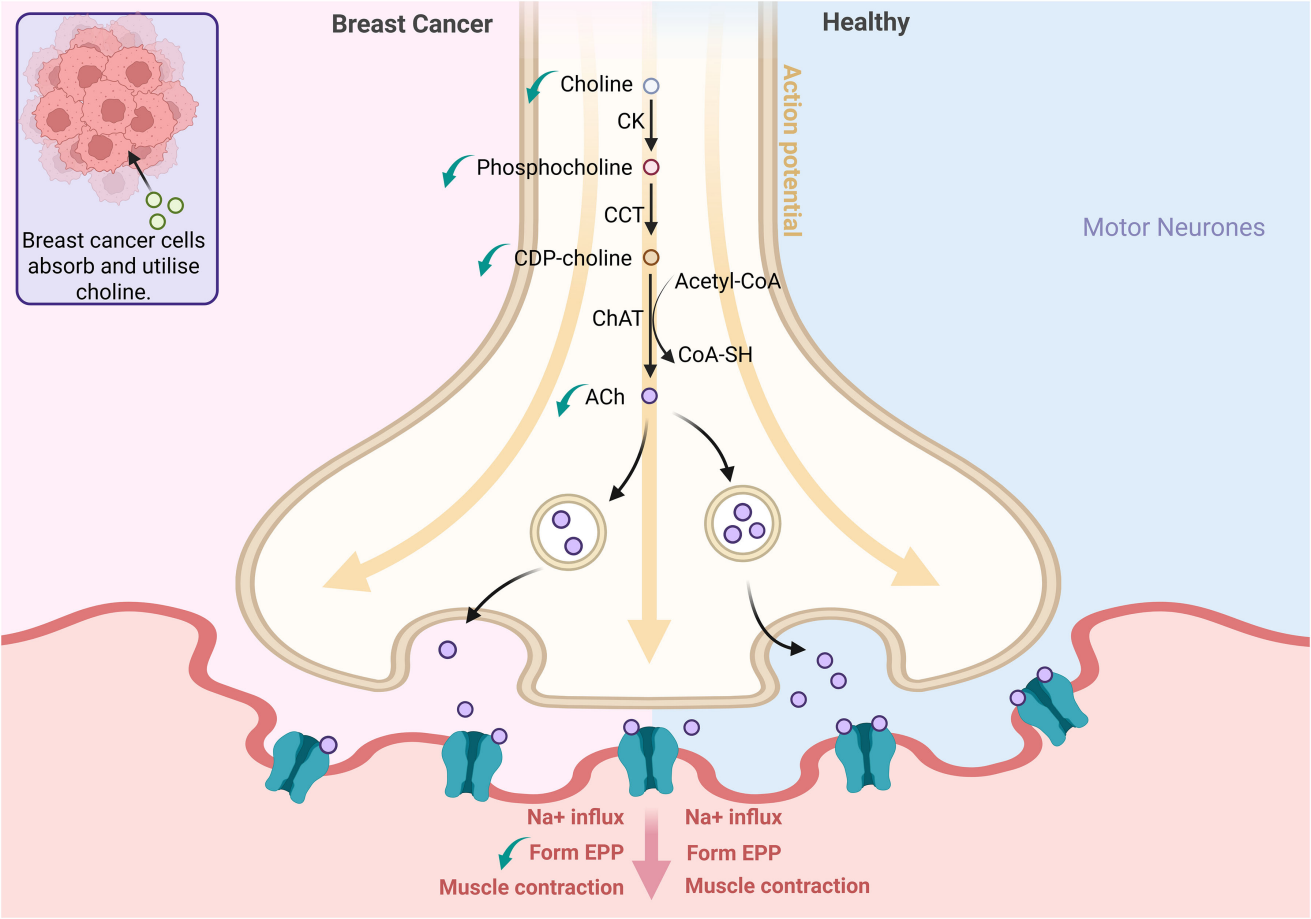
Schematic illustration of the dysfunction of the neuromuscular junction under breast cancer contributing to the development of CRF. Breast cancer cells take up and utilize a large amount of choline to maintain their high metabolic demand, and the reduction of available choline at the neuromuscular junction ultimately leads to a reduction in acetylcholine (Ach) synthesis, resulting in abnormal signaling and contributing to the development of CRF in breast cancer. Creatine kinase (CK), end-plate potential (EPP), phosphocholine cytidylyltransferase (CCT). Created in https://BioRender.com.

Choline, a substrate necessary for breast cancer cell proliferation, is catalyzed by choline kinase (CK) to produce phosphorylcholine, which is subsequently used to synthesize phosphatidylcholine, the primary component of cell membranes. The competitive uptake of choline by breast cancer cells results in a significant decrease in circulating phosphocholine levels (76). This depletion impairs NMJ signaling, disrupting central control of skeletal muscle function and contributing to CRF. This metabolic imbalance suggests that the tumor’s consumption of cholinergic substrates may represent an early step in the impairment of neuromuscular signal transduction (**Figure 2**).

There is increasing evidence that impaired neuromuscular signaling in breast cancer is closely associated with lipid metabolism. Lipids are essential for maintaining cellular structure, energy supply, and various signaling processes. Lipid metabolism, which includes both catabolic processes that generate energy and anabolic processes that synthesize various lipids, is critical for normal cellular function (77). Lipid rafts, nanoscale assemblies of sphingolipids, cholesterol, and proteins, form stable platforms that participate in membrane signaling and transport (78). Disturbances in sphingolipid metabolism, a key component of lipid rafts, can impact NMJ synaptic vesicle release and postsynaptic membrane signaling, leading to dysfunctional NMJ signaling. Several studies have reported abnormalities in sphingolipid metabolism in breast cancer patients, such as decreased phosphatidylcholine levels, increased lysophosphatidylcholine levels, significantly reduced sphingolipid levels, and imbalances in sphingolipid/ceramide ratios (79, 80). These alterations in membrane lipid components contribute to structural damage at both pre- and postsynaptic membranes, affecting NMJ signaling efficiency (81, 82). This damage to the cascade from transmitter synthesis to synaptic transmission forms a “conduction bottleneck,” hindering the conversion of nerve impulses into muscle contraction signals. Ultimately, this results in reduced efficiency of excitation-contraction coupling and contributes to dysfunctional neuromuscular signaling in breast cancer patients (83).

### 3.3 Muscle dysfunction under breast cancer contributes to fatigue

Muscle function plays a crucial role in maintaining normal physiological activities, including the completion of daily tasks, the maintenance of body posture, and the regulation of metabolic homeostasis. Muscle strength and endurance—two core components of physical performance—are closely linked to the development of CRF. Decreased muscular endurance limits patients’ ability to perform repetitive functional movements and negatively impacts their capacity to complete the physical tasks necessary for daily living (84). Clinical observations have revealed that some breast cancer patients exhibit fatigue-related muscle dysfunction, characterized by reduced muscle strength and endurance. This impairment significantly diminishes their quality of life (33).

#### 3.3.1 Reduced muscle mass under breast cancer contributes to fatigue

Muscle mass is a key factor influencing the degree of fatigue in the human body. As a core component of the locomotor system, a decrease in skeletal muscle quality directly leads to weakened muscle strength and endurance, making daily activities more likely to trigger fatigue (85). Wilson et al. found that breast cancer induces reprogramming of skeletal muscle, leading to a reduction in muscle mass, which significantly exacerbates fatigue symptoms (86). The combined effects of these factors result in muscle loss and reduced mobility in breast cancer patients, further exacerbating fatigue. Additionally, patients’ reduced willingness to engage in physical activity due to fatigue hinders the restoration of muscle mass, creating a vicious cycle. However, unlike the persistent loss of skeletal muscle mass observed in cancer cachexia, muscle mass loss under CRF is less severe. In particular, the loss of lower extremity muscle strength in breast cancer fatigue patients can be reversed through exercise or pharmacologic interventions (87, 88).

3-Methylhistidine (3-MH) is a myosin-specific catabolic product that is released into the cytoplasm when myofibrillar proteins are degraded by proteases (89). Its formation is primarily catalyzed by enzymes and is not further metabolized *in vivo*, making it a reliable marker of muscle protein degradation (90). Metabolomics studies have shown an accumulation of 3-MH in the serum of breast cancer patients, directly reflecting the aberrant activation of the ubiquitin-proteasome system (UPS) (91). This may be due to the elevated levels of inflammatory factors, such as TNF-α, which upregulate the expression of E3 ubiquitin ligases (MuRF1/MAFbx) via the NF-κB pathway, contributing to the ubiquitin-mediated degradation of myosin heavy chains (92) Additionally, Salinas et al. found that serum levels of branched-chain amino acids (BCAAs)—including leucine, isoleucine, and valine—were generally reduced in breast cancer patients compared to healthy controls (93). Among these BCAAs, leucine plays a crucial role in muscle growth. It promotes muscle protein synthesis and enhances mitochondrial respiration in adipocytes by activating the mTOR and AMPK pathways. This dual effect facilitates both muscle gain and fat loss. In breast cancer patients, leucine depletion results in the deregulation of the lysosomal Sestrin2 protein and the inhibition of mTORC1 translocation to the lysosomal membrane, leading to a reduction in the phosphorylation of ribosomal S6K1 (94). This disruption ultimately contributes to a decrease in muscle mass in these patients.

#### 3.3.2 Abnormal energy metabolism in subskeletal muscles in breast cancer contributes to CRF

##### 3.3.2.1 Insufficient supply of energy metabolism substrates in skeletal muscle cells leads to exercise limitations in breast cancer patients

The energy required for muscle contraction is provided by the breakdown of ATP. Under normal conditions, the amount of ATP in muscle cells is sufficient to sustain brief contractions. However, inadequate energy supply to muscle cells is a central aspect of peripheral fatigue development in breast cancer, involving several factors such as mitochondrial dysfunction, reduced substrate availability, and the accumulation of tumor metabolites (95). Mitochondria, as the primary site of ATP synthesis, play a critical role in maintaining ATP production. A decline in mitochondrial function directly reduces ATP production, resulting in weakened muscle contractions and increased fatigue.

To support their rapid proliferation, breast cancer cells preferentially utilize glycolysis for energy, even in the presence of oxygen, a phenomenon known as the Warburg effect. This metabolic shift significantly decreases serum glucose and pyruvate levels (8, 96) while increasing lactate production, a byproduct of anaerobic glycolysis (97). This metabolic abnormality reduces the substrates available for energy metabolism in skeletal muscle cells, impeding ATP production and preventing muscles from meeting the energy demands of daily activities. Metabolomic findings show that serum levels of key intermediates in the tricarboxylic acid (TCA) cycle, such as citrate and succinate, are reduced in triple-negative breast cancer (TNBC) patients (8, 98), indicating disruption of mitochondrial metabolism. This metabolic disruption reduces NADH/FADH_2_ production and inhibits the activity of the electron transport chain, resulting in decreased oxidative phosphorylation efficiency.

**Figure 3:**
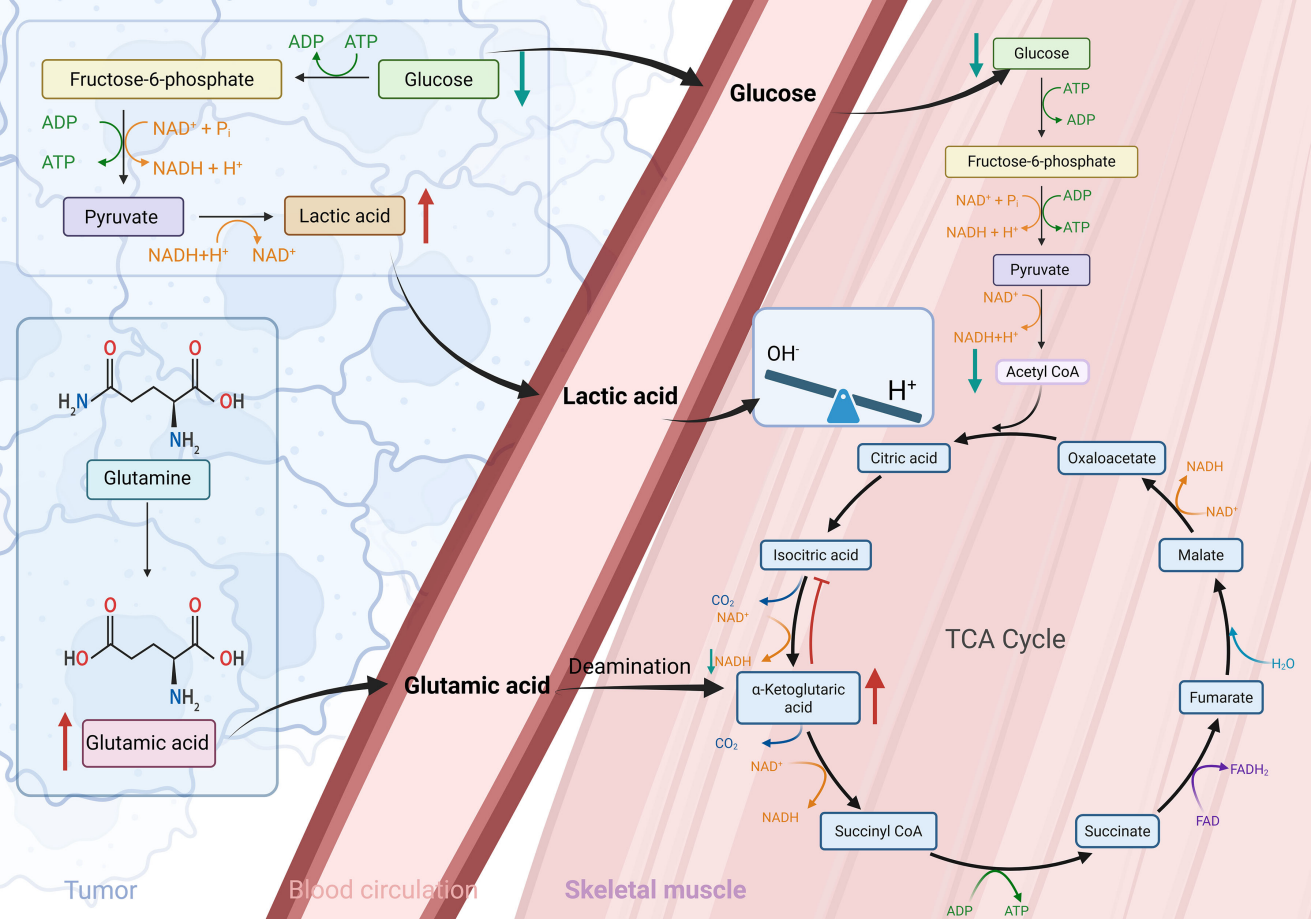
Schematic diagram of the mechanism by which CRF occurs due to insufficient energy supply to skeletal muscle cells under breast cancer. The Warburg effect of breast cancer cells consumes a large amount of glucose and generates lactic acid, leading to the reduction of substrates available for energy metabolism in skeletal muscle cells and the accumulation of lactic acid. A large amount of glutamine is metabolized by breast cancer cells to generate glutamate, which is deaminated to generate alpha-ketoglutarate (α-KG) and enters the tricarboxylic acid (TCA) cycle. The accumulation of α-KG inhibits the activity of isocitrate dehydrogenase through a feedback mechanism, reduces the generation of nicotinamide adenine dinucleotide (reduced form) (NADH), and weakens the oxidative phosphorylation of skeletal muscle cells. Notes: Adenosine diphosphate (ADP), adenosine triphosphate (ATP), flavin adenine dinucleotide (FAD), Flavin adenine dinucleotide (reduced form) (FADH_2_), Carbon dioxide (CO_2_), Nicotinamide adenine dinucleotide (oxidized form) (NAD^+^). Created in https://BioRender.com.

The restricted energy supply to skeletal muscle cells in breast cancer patients may also stem from dysregulated amino acid and nucleotide metabolism. Several metabolomics studies have found significantly elevated levels of glutamine in the serum and tumor tissues of breast cancer patients compared to healthy controls (55, 99, 100). This is due to the tumor’s reliance on glutamine as a source of both carbon and nitrogen to sustain its proliferative needs in the face of limited glucose availability (101, 102). Glutamine is converted by glutaminase into glutamate, which is a key intermediate in the TCA cycle. It is then deaminated to produce α-ketoglutarate (α-KG), which enters the TCA cycle to provide ATP and metabolic intermediates to the cells (103). Elevated serum glutamate levels lead to an accumulation of α-KG, which can inhibit oxidative phosphorylation in skeletal muscle cells. This occurs via a feedback mechanism that inhibits isocitrate dehydrogenase activity, thereby reducing NADH production (104). This also limits the reaction of creatine with ATP, catalyzed by creatine kinase to generate phosphocreatine, impairing the rapid re-synthesis of ATP in skeletal muscle (105). Metabolomic studies on gastrocnemius muscle tissue from TNBC model mice revealed creatine depletion in the gastrocnemius muscle compared to control mice (106). This finding directly reflects the shortage of energy storage and abnormal oxidative phosphorylation in breast cancer, resulting in insufficient energy supply to skeletal muscle cells (**Figure 3**).

Additionally, the accumulation of tumor metabolites such as lactate and ammonia negatively affects muscle endurance (98). Metabolic abnormalities in breast cancer lead to a large accumulation of lactic acid and ammonia in muscle tissue, disrupting the acid-base balance within skeletal muscle and inhibiting key glycolytic enzymes and ATPase activity. Moreover, ammonia accumulation depletes TCA cycle intermediates through transamination, further impeding TCA cycle metabolism (107). These combined factors lead to muscle weakness, reduced capacity for daily activities, and heightened fatigue, significantly impacting patients’ quality of life and physical function.

Abnormal purine metabolism also contributes to muscle dysfunction in breast cancer. Accumulation of hypoxanthine and adenosine in the plasma and tumor tissues of breast cancer patients suggests accelerated ATP degradation and impaired ATP resynthesis (108, 109). These multidimensional metabolic imbalances result in severe depletion of ATP, the “cellular energy currency.” Ubiquinone, also known as coenzyme Q10, is a key component of the mitochondrial electron transport chain that drives cellular energy production. It plays a critical role in transferring hydrogen atoms and electrons within the mitochondrial respiratory chain and is essential for ATP synthesis. Metabolomic analysis of plasma from breast cancer patients revealed alterations in the ubiquinone pathway and other terpenoid quinone biosynthesis pathways (43). A deficiency or dysfunction in ubiquinone may impair mitochondrial energy metabolism and reduce ATP synthesis, further limiting cellular energy supply (110).

##### 3.3.2.2 Decreased muscular endurance due to hematopoietic abnormalities

Exercise-induced robust elevation in skeletal muscle perfusion is governed by local metabolic needs, given the high energy expenditure of sustained muscle contraction; accordingly, regional blood flow is dynamically matched to muscular metabolic demand via circulatory redistribution to optimize peripheral oxygen delivery under constrained cardiac output (111). Rapid proliferation and acidified metabolism within breast cancer tissue establish a hypoxic tumor microenvironment that impairs erythropoiesis and lowers circulating hemoglobin (Hgb) in patients (112), attenuating systemic oxygen transport and inducing intramuscular hypoxia. Restricted intramuscular ATP reserves necessitate immediate activation of supplementary ATP-generating pathways, including phosphocreatine breakdown and intramuscular glycogenolysis to fuel substrate-level anaerobic phosphorylation and aerobic oxidative phosphorylation relying on carbohydrate- and lipid-derived reducing equivalents (113). Chronic hypoxia drives fast-to-slow muscle fiber transformation, compromises contractile function and exercise endurance, elevates myocytic anaerobic glycolysis to provoke lactate accumulation-associated myalgia and fatigue, and damages mitochondrial bioenergetics to suppress ATP production and worsen metabolic insufficiency (114). Breast cancer patients experiencing this state often exhibit easy fatigue, decreased exercise capacity (e.g., difficulty climbing stairs, reduced walking distance), and, in severe cases, symptoms of muscle weakness, feeling excessively tired even during low-intensity activities (115).

Consistent with this finding, plasma metabolomic profiling from breast cancer patients reveals lowered hemoglobin (Hgb) reduces blood oxygen-carrying capacity, forcing myocytes to upregulate glycolysis. Enhanced glycolysis accelerates pyruvate depletion and lactate buildup (98); accumulated lactate suppresses sarcoplasmic reticulum Ca²^+^ release and evokes myalgia via activating acid-sensitive ion channels, alongside diminished myosin ATPase activity to impair skeletal muscle contractility and exacerbate fatigue (116). Nguyen et al. found elevated levels of adenosine (ADO) in breast cancer patients compared to healthy volunteers (8), indicating impaired mitochondrial oxidative phosphorylation in the context of breast cancer, which limits ATP regeneration. In this hypoxic state, ATP catabolism is promoted, leading to the dephosphorylation of large amounts of AMP to produce ADO. ADO can dilate blood vessels by activating the A_2_A receptor, increasing blood flow to skeletal muscle, and helping to counteract the insufficient “energy flux” under tissue hypoxia (117). Although this alteration enhances oxygen supply to skeletal muscle tissues, it accelerates substrate depletion, leading to inadequate energy supply during the later stages of endurance exercise, causing patients to exhibit symptoms of CRF.

#### 3.3.3 Chemotherapy induces abnormalities in skeletal muscle metabolism in breast cancer patients

Chemotherapy is a common component of breast cancer treatment regimens, with anthracycline- and cyclophosphamide (CTX)-based therapies frequently used as adjuvant treatments for early-stage breast cancer (118). CTX is metabolized to amide nitrogen mustard, acrolein and other metabolites that bind to DNA in rapidly dividing cancer cells and exert potent cytotoxicity. By contrast, anthracyclines suppress tumor proliferation via disrupting DNA replication, arresting cell cycle, triggering iron-dependent lipid peroxidation to modulate cellular redox homeostasis, and inhibiting topoisomerase II activity (119).While these chemotherapeutic agents are effective in inhibiting cancer cell proliferation, they also produce adverse effects, with CRF being a common side effect of chemotherapy (120). Notably, CTX-induced skeletal muscle toxicity primarily contributes to muscle dysfunction by triggering myocyte apoptosis, disrupting muscle repair processes, and causing structural damage to muscle tissue, ultimately compromising physiological performance (121). Joris et al. reported that a single dose of CTX administered over four days resulted in skeletal muscle atrophy and mitochondrial alterations in breast cancer patients (122). These changes included reduced mitochondrial function and quantity, impaired mitochondrial dynamics, and increased apoptosis, all of which are mediated by complex alterations in signaling pathways that affect muscle cell activity and proliferation. Both CTX and anthracycline therapies lead to muscle mass loss through mitochondrial dysfunction and apoptosis-activated pathways, providing evidence for the mechanisms underlying abnormal skeletal muscle function resulting from breast cancer chemotherapy.

Metabolomic analysis by Caridad et al. of serum samples from patients receiving neoadjuvant chemotherapy with anthracyclines in combination with CTX found an increase in lysophospholipids from baseline pre-chemotherapy levels, while carnitine levels exhibited a trend toward decrease (123). CTX’s property of inhibiting the division and proliferation of normal hematopoietic cells can damage the hematopoietic system, leading to a reduction in the number of nucleated cells in the bone marrow and decreased hematopoietic activity. The vertebrate spleen plays a crucial role in hematopoiesis, and studies have demonstrated that extramedullary hematopoiesis in the spleen is enhanced in animal models of breast cancer (124). Tian et al. found that choline levels were reduced in metabolomic studies of spleen tissues from CTX-treated 4T1 tumor-bearing mice (125). Choline is essential for the synthesis of phospholipids, which are critical components of cell membranes. Disruption of choline metabolism not only affects NMJ delivery efficiency, as discussed in section “3.2,” but also impacts the normal function of iron transport proteins in cell membranes. A choline deficiency can impair iron transport, leading to iron homeostasis imbalances, reduced oxygen transport efficiency, and subsequently decreased availability of oxygen for ATP production in skeletal muscles. This results in diminished skeletal muscle endurance in breast cancer patients.

## 4 The role of TCM interventions in breast cancer-related fatigue

Currently, treatment options for malignant tumors are increasingly diverse. Relying on a single treatment method often fails to meet patients’ needs for survival and quality of life. Furthermore, prolonged use of chemotherapeutic drugs can lead to the development of drug resistance, subsequently diminishing their therapeutic efficacy (126).In contrast, TCM, with its unique compounding theory and natural drug properties, offers advantages such as multi-targeting, reduced toxicity, and fewer side effects. TCM has the potential to alleviate the toxic side effects associated with breast cancer treatments while enhancing therapeutic effectiveness. Therefore, it shows considerable promise as a long-term adjuvant therapy and alternative treatment for CRF in breast cancer patients (20).

### 4.1 The role of traditional Chinese medicine compounding and proprietary medicine interventions on breast cancer-related fatigue

Fufang E’jiao Jiang (FEJ) is a patented compound TCM marketed in China, composed of *Colla Corii Asini*, *Red Ginseng*, *Radix Rehmanniae Praeparata*, *Radix et Rhizoma Ginseng*, and *Fructus Crataegi*. *Colla Corii Asini* is the primary active ingredient, and FEJ is commonly used to treat symptoms of qi and blood deficiency in CRF. It has demonstrated significant clinical efficacy and safety. Studies have shown that FEJ intervention can improve patients’ behavioral, emotional, sensory, and cognitive performance. It alleviates symptoms such as pain, fatigue, depression, anxiety, and drowsiness, and enhances emotional, functional, and fatigue-related quality of life (127, 128). Liu et al. investigated FEJ in a radiotherapy- and chemotherapy-induced bone marrow transplantation mouse model and found that FEJ promoted the recovery of bone marrow hematopoietic function by improving the bone marrow hematopoietic microenvironment, stimulating cell proliferation, preventing apoptosis of bone marrow nucleated cells, and enhancing the expression of IL-1β, IL-3, IL-6, SCF, and granulocyte-macrophage colony-stimulating factor, while inhibiting the expression of TGF-β (129).

Jingfang Granules (JFG) is a modern TCM compound formulation containing *Schizonepetae Herba*, *Saposhnikoviae Radix*, *Notopterygii Rhizoma et Radix*, *Angelicae Pubescentis Radix*, *Bupleuri Radix*, *Peucedani Radix*, *Chuanxiong Rhizoma*, *Aurantii Fructus*, *Poria*, *Platycodonis Radix*, and *Glycyrrhizae Radix et Rhizoma*. Wang et al. found that JFG activates the tricarboxylic acid (TCA) cycle and promotes ATP production by inducing isocitrate dehydrogenase expression. This provides essential energy for mice with chronic fatigue syndrome. Additionally, the elevated expression of isocitrate dehydrogenase inhibited the NLRP3 inflammasome signaling pathway and NF-κB signaling, while activating the antioxidant Nrf2/HO-1/NQO1 signaling axis, thereby reducing inflammation (130).

Shenqi Fuzheng Injection (SFI) is a modern TCM compound preparation composed of active ingredients from *Astragali Radix* and *Codonopsis Radix*. Researches have shown that SFI can significantly alleviate fatigue-like behaviors in malignant mouse models by targeting skeletal muscle energy metabolism regulation. Guo et al. used integrated network pharmacology and untargeted metabolomics to find that SFI effectively activates the mitochondrial respiratory chain function in skeletal muscle, promoting HIF-1α-mediated autophagy of muscle mitochondria (131). By optimizing the expression of key enzymes in the TCA cycle, SFI improves ATP synthesis efficiency. These findings suggest that SFI is an effective intervention for alleviating CRF by addressing the vicious cycle of “mitochondrial dysfunction-ATP depletion” (132).

Danggui Buxue Decoction (DBD) is a TCM decoction consisting of *Angelicae Sinensis Radix* and *Astragali Radix*, primarily used to tonify qi and promote blood production. Miao et al. found that DBD significantly influenced the biosynthesis of phenylalanine, tyrosine, and tryptophan, as well as the metabolism of glycine, serine, threonine, glycerolipids, glyoxylates, and dicarboxylates in rats with a chronic fatigue syndrome model (133). Additionally, Liu et al. demonstrated that DBD had a protective effect against CTX chemotherapy-induced cardiotoxicity in mice, significantly inhibiting serum levels of alanine aminotransferase, aspartate aminotransferase, creatine kinase, and lactate dehydrogenase. These results suggest that DBD holds great potential for treating CRF in breast cancer patients.

### 4.2 The role of TCM monomers and active ingredient interventions on breast cancer-related fatigue

In recent years, extensive research has been conducted on the biological properties and pharmacological effects of natural drug extracts from *Curcumae Longae Rhizoma*. Curcumin, a major constituent of turmeric rhizomes, has been shown to possess anti-inflammatory, antioxidant, and antiproliferative properties against breast cancer cells (134). Zhang et al. found that curcumin regulates pyruvate metabolism, glycolysis, and dicarboxylic acid metabolic pathways, reduces the expression of inflammatory factors, inhibits the activation of the NF-κB pathway, and decreases the ubiquitylation levels in muscle tissues. These effects help alleviate mitochondrial dysfunction, improve muscle tissue malnutrition and energy metabolism, and slow the loss of body weight and gastrocnemius index (106). Additionally, curcumin acts as a free radical scavenger and promotes the synthesis of the endogenous antioxidant glutathione (GSH), which protects cells from oxidative damage. *In vitro* and animal studies have shown that curcumin enhances superoxide dismutase activity and increases cellular and serum GSH levels, demonstrating specific antioxidant effects (135). These findings suggest that curcumin can alleviate fatigue in breast cancer patients through its anti-inflammatory and antioxidant properties.

Both ethanolic and aqueous extracts of *Cordyceps militaris*, an entomopathogenic fungus, have shown anticancer, anti-fatigue, and anti-hypoxic activities. Previous reports indicate that the ethanol crude extract of Cordyceps significantly reduces the proliferation of breast cancer cells and promotes apoptosis (136). Oh et al. found that the ethyl acetate fraction of Cordyceps militaris (CM-EA) significantly increased the swimming endurance and reduced fatigue in tumor-bearing mice. Metabolomic analyses revealed increased succinic acid levels and decreased lactic acid levels in the liver tissues of mice, suggesting that CM-EA improves CRF by activating the TCA cycle and inhibiting glycolysis (137).

*Ginseng*, a renowned traditional Chinese medicine, plays a crucial role in treating qi deficiency, a common clinical condition in breast cancer patients. In TCM, spleen deficiency leads to insufficient qi and blood, causing muscle malnutrition and symptoms such as fatigue and lethargy. Li et al. found that ginseng intervention improved immune function in rats with spleen qi deficiency by modulating the metabolism of arginine, pantothenic acid, coenzyme A, thiamine, taurine, and tryptophan (138). *Ginsenoside Rd*, a key component of ginseng, has neuroprotective effects through anti-inflammatory, antioxidant, anti-apoptotic mechanisms, and regulation of calcium ion (Ca²^+^) levels. It also modulates the NF-κB, PI3K, and MAPK signaling pathways (139). Ding et al. demonstrated that ginsenoside Rh2 inhibited proliferation and promoted apoptosis in triple-negative breast cancer (TNBC) cells by targeting the IL-6/JAK2/STAT3 pathway (140). Ginsenoside Rg1, another natural compound, has been shown to exert anti-fatigue effects by modulating the epidermal growth factor receptor and influencing taurine and mannose 6-phosphate metabolism (141). These results suggest that ginsenosides have significant potential in alleviating CRF in breast cancer patients through neuroprotective and antioxidant effects.

*Bupleuri Radix* is well known for its anti-inflammatory properties, and its main active ingredient, saikosaponin (SS), has demonstrated anti-inflammatory bioactivity. Ma et al. used a metabolomics approach to show that SS exerts its anti-inflammatory effects by regulating nicotinic acid, nicotinamide, and arachidonic acid metabolism. Saikosaponin D (SSD) has been identified as the most potent anti-TNBC compound, inducing apoptosis in TNBC cell lines by inhibiting the Wnt/β-catenin signaling pathway (142). SSD also reduces pro-inflammatory cytokines and enhances antioxidant enzyme activities through the PI3K/AKT/Nrf2 pathway, improving muscle atrophy and alleviating fatigue symptoms in mice (143, 144). Additionally, quercetin, a polyphenolic flavonoid present in Bupleuri Radix, has strong antioxidant activity. Quercetin has been shown to reduce lactic acid accumulation in the tumor microenvironment by inhibiting anaerobic glycolysis in breast cancer cells and decreasing lactate transporter protein expression (145, 146). Zhang et al. found that quercetin enhanced the therapeutic effect of adriamycin combined with CTX and reduced CTX-induced cardiotoxicity in TNBC cells (147). Wu et al. demonstrated that quercetin improved physical endurance in fatigued mice by altering energy metabolism, as evidenced by metabolomic analyses (148). These findings suggest that quercetin can influence the development of CRF by inhibiting oxidative stress and lactate production in breast cancer cells.

**Figure 4:**
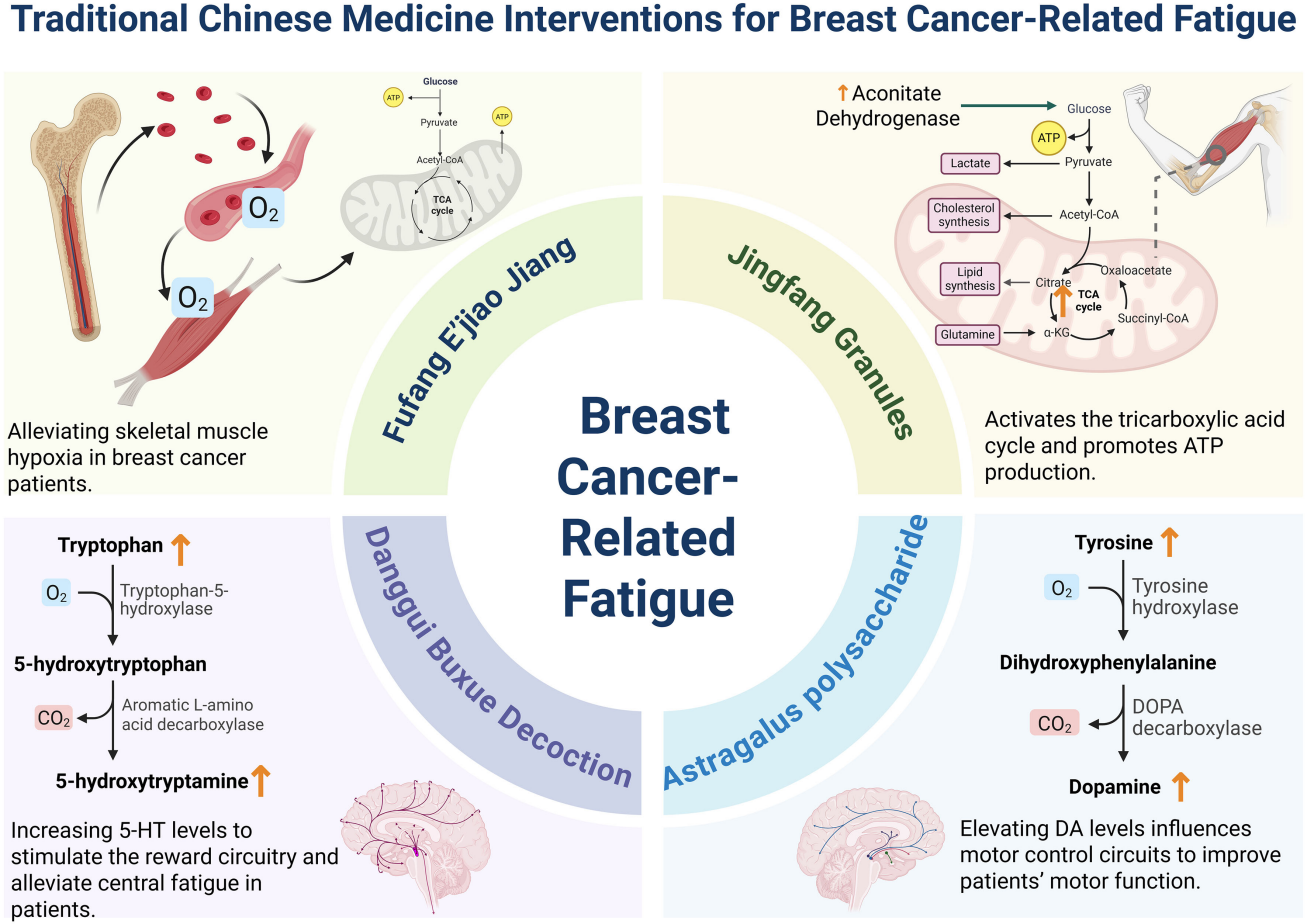
Schematic diagram of the effects of representative traditional Chinese medicine compound prescriptions and active components of traditional Chinese medicine on the metabolomics of patients with breast cancer-related fatigue. Oxygen (O_2_), L-3,4-dihydroxyphenylalanine (DOPA). Created in https://BioRender.com.

*Astragali Radix* is widely used in TCM for treating qi deficiency and fatigue. Its main active ingredient, astragalus polysaccharide, has immunomodulatory, anti-tumor, and antioxidant effects. Clinical data show that astragalus polysaccharide combined with anthracycline chemotherapy can reduce fatigue, insomnia, and negative emotions, while improving overall health, particularly in premenopausal breast cancer patients (149, 150). Wei et al. conducted metabolomics analysis on the serum of chronic fatigue mice and found that astragalus polysaccharide improved tyrosine metabolism, alleviated cognitive deficits, and reduced fatigue symptoms by regulating the disrupted tyrosine metabolism pathway (151) (**Figure 4**). 

Although the aforementioned traditional Chinese medicine formulations and active ingredients have demonstrated multi-targeted and low-toxicity advantages in alleviating cancer-related fatigue associated with breast cancer, existing research remains predominantly limited to animal studies and small-scale clinical observations, with a lack of high-quality clinical evidence from large-scale, randomised, placebo-controlled trials. Most studies report only qualitative results, such as improvements in fatigue scores, and lack objective quantitative indicators such as changes in metabolite concentrations, rates of ATP elevation, rates of mitochondrial function improvement, and rates of muscle protein synthesis. Furthermore, the pharmacological basis of TCM formulations remains unclear, and the influence of numerous factors—such as dose-response relationships, duration of administration, and stratification of indications based on breast cancer subtypes and treatment stages—has not yet been systematically elucidated. Regarding mechanism validation, no direct causal evidence linking metabolomic biomarkers to therapeutic efficacy has been established, with research remaining limited to pathway associations. Crucially, most existing intervention studies on TCM and its active ingredients rely solely on a ‘phenotypic improvement’ validation model, failing to adopt metabolomic biomarkers as core endpoints for efficacy evaluation. Consequently, it remains unclear which metabolites can serve as response markers, severely limiting the precision and standardisation of TCM interventions for breast cancer-related fatigue.

## 5 Discussion

Unlike previous reviews that have explored the pathogenesis of CRF, this paper focuses on summarizing the results of existing metabolomics studies and discusses the pathogenesis of breast cancer-related fatigue from three key perspectives: dysregulation of central neurotransmitter networks, disrupted NMJ signaling, and decline in peripheral skeletal muscle function. Additionally, we summarize the effects of TCM interventions on breast cancer-related fatigue, categorizing them into TCM combinations, TCM monomers, and active ingredients.

However, several gaps remain in the current research on CRF in breast cancer, and this review has some limitations in exploring both the pathogenesis of CRF and the application of TCM. Firstly, there is a clear lack of cross-study validation, most studies did not classify breast cancer subtypes in the patient population, which may lead to bias in identifying differential metabolites, for example, whilst reports of reduced tryptophan levels are consistent, variations in glutamate and glutamine levels are observed due to differences in samples and detection methods (55, 97, 101, 102). Secondly, the lack of longitudinal studies tracking metabolic trajectories before and after treatment in breast cancer patients limits the ability to analyze changes in their metabolic profiles with respect to therapeutic regimens. Third, most metabolomics studies have focused on peripheral blood, breast tissue, excretions, and *in vitro* cultured breast cancer cell lines. These sample types cannot directly capture changes in central nervous system neurotransmitters, whereas tissue metabolomics provides insights into the underlying mechanisms, but is invasive and unsuitable for longitudinal monitoring. As a result, it is difficult to directly and accurately explain the central metabolic disturbances underlying CRF in breast cancer. Furthermore, current research on the mechanisms of action of TCM combinations and monomers is still underdeveloped. There is a need for more exploration into the synergistic effects of multi-target combination preparations and their mechanisms. Moreover, differences between animal models and clinical practice, along with the lack of support from prospective multicenter studies, hinder the validation of metabolic markers. Importantly, this review has not fully elucidated the direct causal relationship between specific metabolites and fatigue symptoms, nor have we analyzed the interaction network between multiple metabolic pathways and their dynamic regulatory mechanisms. These shortcomings highlight the need for future studies to focus on analyzing the specificity of each breast cancer subtype, employ multidimensional bioinformatics, enhance dynamic monitoring data, and strengthen the study of metabolic networks regulated by traditional Chinese medicines. Such efforts will help advance the development of precision metabolic medicine for CRF in breast cancer.

## 6 Future perspective

Metabolomics research offers unique advantages compared to other related fields, particularly in its ability to directly reflect the metabolic status of the tumor microenvironment and blood circulation. This capability provides new insights into the metabolic alterations occurring in the macroscopic environment under breast cancer conditions. Additionally, metabolomics shares commonalities with tumor genomics and proteomics, all of which aim to elucidate the molecular mechanisms underlying breast cancer and the development of associated fatigue (152). The intersection of these fields creates a systems biology framework for studying the pathogenesis of breast cancer and related fatigue, facilitating a comprehensive understanding of the nature of cancer-related fatigue.

Looking ahead, research into the mechanisms underlying breast cancer-related fatigue should prioritize large-scale, multicenter, longitudinal metabolomic studies that encompass various breast cancer subtypes and treatment stages. The goal is to capture dynamic metabolic changes before, during, and after treatment, thus distinguishing alterations caused by the cancer itself from those induced by treatment. Establishing a stable and reproducible set of metabolic biomarkers for diagnosing and monitoring treatment response in breast cancer-related fatigue is essential. To achieve this, further studies should employ multi-omics integration strategies that combine metabolomics with genomics, proteomics, and gut microbiome analysis. Such integrated approaches are crucial for uncovering the systematic networks underlying chronic fatigue syndrome rather than focusing solely on isolated pathways. Additionally, research in traditional Chinese medicine should transition from descriptive metabolomic analysis to mechanism-based validation. This involves utilizing genetic or pharmacological interventions to confirm the causal roles of specific metabolites and developing metabolic biomarkers for patient stratification, ultimately enabling personalized TCM interventions. Advances in bioinformatics and machine learning will support the identification of metabolite combinations capable of predicting treatment outcomes. This shift aims to transform the management of breast cancer-related fatigue into a precision medicine model. In summary, a comprehensive and integrated research approach is essential for unraveling the complexities of breast cancer-related fatigue, enhancing the potential for targeted therapeutic strategies and improved patient outcomes.
